# Global mapping of stratigraphy of an old-master painting using sparsity-based terahertz reflectometry

**DOI:** 10.1038/s41598-017-15069-2

**Published:** 2017-11-08

**Authors:** Junliang Dong, Alexandre Locquet, Marcello Melis, D. S. Citrin

**Affiliations:** 1 School of Electrical and Computer Engineering, Georgia Institute of Technology, Atlanta, GA 30332-0250 USA; 2 UMI 2958 Georgia Tech-CNRS, Georgia Tech Lorraine, 2 Rue Marconi, Metz, 57070 France; 3 Profilocolore srl, Via Spluga, Roma, 22 00 141 Italy

## Abstract

The process by which art paintings are produced typically involves the successive applications of preparatory and paint layers to a canvas or other support; however, there is an absence of nondestructive modalities to provide a global mapping of the stratigraphy, information that is crucial for evaluation of its authenticity and attribution, for insights into historical or artist-specific techniques, as well as for conservation. We demonstrate sparsity-based terahertz reflectometry can be applied to extract a detailed 3D mapping of the layer structure of the 17th century easel painting *Madonna in Preghiera* by the workshop of Giovanni Battista Salvi da Sassoferrato, in which the structure of the canvas support, the ground, imprimatura, underpainting, pictorial, and varnish layers are identified quantitatively. In addition, a hitherto unidentified restoration of the varnish has been found. Our approach unlocks the full promise of terahertz reflectometry to provide a global and detailed account of an easel painting’s stratigraphy by exploiting the sparse deconvolution, without which terahertz reflectometry in the past has only provided a meager tool for the characterization of paintings with paint-layer thicknesses smaller than 50 *μm*. The proposed modality can also be employed across a broad range of applications in nondestructive testing and biomedical imaging.

## Introduction

Studies at the intersection between the quantitative sciences and the humanities have been especially fruitful in the last few decades with the availability of new analytic tools that permit noninvasive studies of art. In particular, physical characterization of art using emerging tools may provide information that conclusively confirms or refutes judgments based on connoisseurship. Determination of the physical characteristics, not only across a painting but also in depth, is one of the most important procedures to gain insight into its structure. The layer-by-layer structure, or stratigraphy, reveals the sequential application of the preparatory and ground layers on the support (e.g., canvas or wood panel), pictorial layers, and varnish, as well as of possible subsequent revisions or restorations. A detailed knowledge of the stratigraphy provides a basis for evaluation of its authenticity and attribution, insight into historical or artist-specific techniques for art-historical studies, as well as the recognition of any decay and of consequent conservation and/or restoration requirements. For paintings, stratigraphic analysis can reveal the process in which the paint layers are applied, and consequently, to unveil how the artist worked. The conventional approach to obtain information on the stratigraphy is based on the characterization of the cross-sections of micro-samples taken from the objects with standard micro-analytical tools, such as visible-light and electron microscopy, energy-dispersive X-ray spectroscopy, Raman and infrared spectroscopy^1^. This approach is invasive, resulting in the destruction of the integrity of the painting.

Various noninvasive and noncontact modalities which can provide *in*-*situ* quantitative information in depth, such as confocal x-ray fluorescence (with elemental distribution contrast)^2^, femtosecond pump-probe microscopy (with molecular and structural constrast)^3^, nuclear magnetic resonance (NMR) (with ^1^H abundance constrast)^4^, and optical coherence tomography (with structural constrast)^5^, are under active research. Nonetheless, to date, the aforementioned optical techniques, as well as x-ray fluorescence (the emitted photon is optical) have been limited to demonstrations permitting measurements to depths up to only tens or ~200 *μ*m due to strong optical attenuation, while NMR has only been used to demonstrate several Z-scans across a painting^6^. These methods suffer from a set of overlapping difficulties for the task at hand, namely the global mapping of the stratigraphy of art paintings, including limited penetration as well as the unsuitability of sub-micrometric scale probes to provide the desired information on the 10-*μm*-to-*mm* scales in depth and the 100-*μm*-to-*m* scales in the transverse direction. In summary, while these techniques in some instances provide high-resolution depth information about paintings, they have not been demonstrated to enable access to produce a *global* 3D mapping of the stratigraphy of a painting.

Due to the penetrative capability of terahertz (THz) electromagnetic radiation in broad classes of nonmetallic material^7^, THz reflectometry based on THz time-domain spectroscopy (THz-TDS) systems has attracted considerable interest for revealing the stratigraphy *in situ*, as well as hidden features of art paintings^8^, such as the artist’s signature^9^, under-drawings^10^, and modifications due to the reuse of an earlier painting or canvas^11^. THz reflectometry may provide information in depth by analyzing the reflected THz signal with an incident approximately single-cycle THz pulse (which means that the spectrum of the THz radiation is broadband-from ~100 GHz up to ~3 THz for a typical THz-TDS system)^12^. Due to dielectric discontinuities with depth associated with the various paint and other layers in the painting, reflected temporal THz echoes associated with the Fresnel coefficients at various interfaces are recorded as a function of transverse position in amplitude and time delay^13^. The stratigraphy of paintings can be reconstructed by precise extraction of THz echo parameters from the reflected THz signal. So far, some panel paintings and wall paintings have been studied by THz reflectometry, in which certain details of the stratigraphy have been revealed^14–17^. One lacuna in the past success of THz stratigraphic characterization of paintings, however, is the field of pre-19th century easel paintings^18^, where the paint layer thicknesses are usually smaller than 50 *μ*m^19^ (though paint-layer thickness varies by artist and style even throughout this period). This characteristic paint-layer thickness is optically thin in the THz regime, since it is much less than the time over which the THz pulse propagates within its duration, corresponding to the depth resolution of a typical THz-TDS system^20^. Nonetheless, there may be spectral information present at the relevant short wavelengths that is obscured in the raw signal. In the context of THz reflectometry, when dealing with optically thin paint layers, the THz echoes resulting from the various interfaces between layers will partially or even totally overlap in time and thus these echoes will merge rather than be distinct. Consequently, to our knowledge, the detailed stratigraphy of pre-19th century easel paintings has not been clearly revealed by THz reflectometry, as the paint layers in easel paintings are frequently very thin in the THz regime, especially for the 16th and 17th century easel paintings.

Deconvolution, which can yield sub-wavelength and sub-pulse-width depth resolution^21^, has shown great potential in resolving the overlapping echoes and characterizing the stratigraphy. The resulting impulse-response function provides a contrat mechanism for producing images that ideally depends on the sample structure rather than is dominated by characteristics of the probe itself. THz frequency-wavelet domain deconvolution (FWDD) has been specifically designed and has been applied to enhance the depth resolution and characterize multilayered structures with optically thin layers in the THz regime^22,23^. However, the depth resolution achieved by FWDD appears not to be sufficiently high to characterize the stratigraphy of typical easel paintings before the 19th century. In this study, in order to tease out the detailed stratigraphic information that is actually contained in the reflected THz signal, the sparse representation is introduced in the deconvolution process. Generally, the reflected THz signals from multilayered structures are a class of very special signals comprised of a limited number of echoes; therefore, the corresponding impulse-response functions have a sparse representation, which means that only a limited number of data points have non-zero values. This feature enables us to exploit the sparse constraint and retrieve the impulse-response function by sparsity-based time-domain deconvolution. Here we present THz reflectometry with subwavelength depth resolution for stratigraphic characterization based on a sparsity-based time-domain deconvolution algorithm as developed by our group^24^, which enables us to show the detailed stratigraphy of a 17th century easel painting based on THz reflectometry for the first time to our knowledge. The paint layers on the canvas support of this painting are significantly thinner than the dominant wavelength in the available THz spectrum; thus, the THz echoes reflected from the paint layers on the supporting canvas of this painting are strongly overlapped in the first cycle of the reflected THz signal. With the sparsity-based impulse-response function achieved by deconvolution, we gain 3D quantitative insight into the detailed stratigraphy above the canvas throughout the painting, including the varnish, pictorial layer, underpainting, imprimatura, and the ground layer. In addition, we identify delaminations in the pictorial layer associated with age-induced craquelure, and locate a hitherto unidentified extensive restoration of the painting. We emphasize, this is the first time, to our knowledge THz reflectometry has resolved multiple layers in an easel painting with such thin layers. Although we focus on easel paintings, the proposed modality can be applied to a wide range of culture heritage objects and provides invaluable information for art-historical studies, as well as potentially for conservation, restoration, and authentication.

## Results

### Sample and Experiment

**Figure 1:**
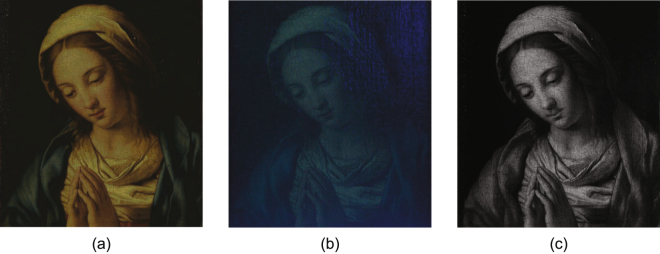
Images of *Madonna in Preghiera*. (**a**) Visible photograph of the *Madonna in Preghiera*; (**b**) Image of *Madonna in Preghiera* obtained by UV Fluorescence; (**c**) Image of *Madonna in Preghiera* obtained by IR Reflectography.

The painting in this study was chosen as a typical painting for the period that is specifically of the type that has resisted considerable attempts to characterize its stratigraphy, *i*.*e*., the relevant layer thicknesses are on the order of tens of microns. It is a 17th century oil painting on canvas, entitled the *Madonna in Preghiera* attributed to the workshop of Giovanni Battista Salvi da Sassoferrato, in the collection of the Musée de la Cour d’Or, Metz--Metz Métropole, France, inventory number 11621, shown in Fig. 1(a). The dimensions of this painting are 24 cm by 32 cm. The canvas is mounted on a wood stretcher. Visually, the paint application itself is smooth and uniform, though subsequently formed craquelure or other inclusions in the canvas give rise to nonuniform features in the surface texture. Multispectral imaging was performed with the Profilocolore HMI (Hypercolorimetric Multispectral Imaging) system, based on a modified Nikon D800FR camera to obtain the spectral reflectance of the surface in the range of 300–1000 nm, with a spatial resolution of 36 Mpixel. The intention is that different types of physical structures and pigments may be revealed by various wavelengths. Images obtained by ultraviolet (UV) fluorescence and infrared (IR) reflectography are shown in Fig. 1(b,c), respectively. UV fluorescence can reveal the presence of natural resin varnishes, which often fluoresce under UV light. It is also able to identify any retouchings on top of an aged varnish, since oil paint and newer varnish do not fluoresce under UV^25^. Retouchings therefore appear as dark patches on the varnish surface. In Fig. 1(b), a piece of dark patch near the *Madonna*’s head can be clearly identified, which may corresponding to a retouching area. IR light can pass through the varnish and reveal the features of the surface and subsurface of the pictorial layers. In Fig. 1(c), age-induced craquelure in the pictorial layer can be observed.

THz reflective imaging based on a typical THz-TDS system (Teraview TPS Spectra 3000) was performed at almost normal incidence from the top (i.e., painted) surface of the painting. Before scanning the painting, a reflected THz reference signal was recorded by setting a metal plate at the sample position. The THz emitter and detector were raster-scanned by a set of motorized stages moving in the *X* and *Y* directions with a 1-mm step size over a 22 cm by 25 cm region of the painting. The painting was mounted paint facing downward supported by the edges of the stretcher in a horizontal configuration. After performing the scan, the positions of the last echo in the reflected signal at each point imaged, corresponding to the bottom surface (interface between the canvas and the air) of the painting, were aligned to the same position temporarily to correct for gravity-induced sag and convenience for further signal processing. After this alignment, a 3D volume raw data set was acquired.

### THz Raw Images

**Figure 2:**
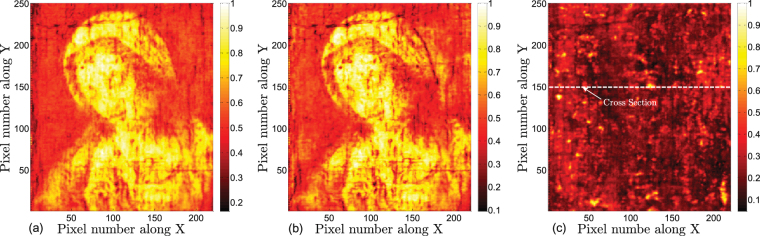
THz C-scans using on the raw THz data based on the following contrast mechanisms in the reflected signal: (**a**) peak-to-valley amplitude; (**b**) integrated spectrum between 0.5 and 1.0 THz; (**c**) peak-to-valley amplitude between 13 to 18 ps. The horizontal line at *Y* = 150, marked ‘cross section’, is in reference to the B-scan in Fig. 3. Contrast is normalized to one.

First, we present the THz images based on the raw THz signals directly obtained from the scan. THz C-scans (two-dimensional presentation of data displayed as a top planar view of the painting) are shown in Fig. 2. For the THz C-scan in the time domain shown in Fig. 2(a), the imaging contrast mechanism chosen is the peak-to-valley amplitude of the reflected THz signal (mainly the peak-to-valley amplitude of the first cycle). The THz C-scan in the frequency domain can also be obtained by taking the Fourier transform of the raw waveform at each pixel, and integrating the magnitude of the frequency components in a given frequency window--between 0.5 and 1.0 THz, in the case of Fig. 2(b). THz C-scans based on these two contrast mechanisms mainly present the THz response of the paint pigments (as we clearly see that the dominant features are those of the visual aspects of the painting), and also reveal the surface roughness, as well as evidence of subsurface features. In Fig. 2(a), the Fresnel coefficient between the top-most paint layer and air depends on the refractive indices of the pigment, and so the image of the *Madonna* is clear. Many of the more irregular features, which will be commented on in depth below, are associated with the surface morphology of the painting. In the presence of a surface irregularity, enhanced scattering of THz radiation ensues, and consequently there is a weaker specular signal. In a similar fashion, Fig. 2(b) reveals similar (but not identical) features. In fact, the THz C-scan in the frequency domain is somewhat more effective in revealing surface features, as higher frequency components within the integrated spectral bandwidth, correspond to shorter wavelengths, bringing out small and subtle features.

**Figure 3:**
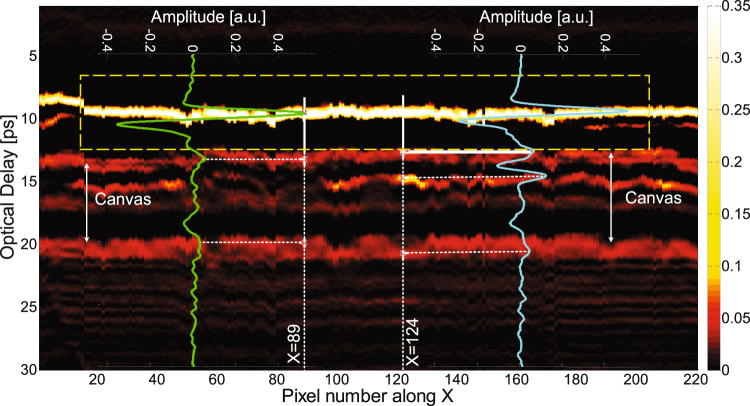
The THz B-scan based on the amplitude of raw signals with the cross-section *Y* = 150. Two typical THz reflected signals are plotted corresponding to the THz B-scan, with the waveform at pixel (89, 150) in green and the waveform at pixel (124, 150) in blue.

While a THz C-scan is an *X*,*Y* image of the painting, a THz B-scan (two-dimensional presentation displayed as a cross-sectional view of the painting) provides depth information along a line in the *X*,*Y* plane. Note that optical delay in the received reflected signal serves as a surrogate for depth. Translating from optical delay to depth also requires a knowledge of the refractive indices of the various layers, which are assumed not to vary significantly within the THz spectral bandwidth. The THz B-scan based on the raw data with the cross-section *Y* = 150 is plotted in Fig. 3 to show the structure of the painting. Note that in the present case, the contrast mechanism is simply the magnitude of the reflected signal at a given optical delay. The raw THz reflected signals at pixels (89, 150) and (124, 150) are also plotted for reference. These two signals show similar features associated with reflections off corresponding features, although there are slight differences due to local variations across the painting. The roughly horizontal features in the B-scan indicate reflections off interfaces between layers in the painting. The first large feature in optical delay (between 8 and 10 ps optical delay) is due to reflection off the painting surface, below which we see a clear feature (~13 ps) due to the reflection off the ground-layer/canvas interface. The canvas layer can be clearly identified as corresponding to time delays from 13 to 20 ps. The pronounced horizontal features at ~15.5 and 17 ps are due to the fact that the painting contains a double layer of canvas, as is verified by visual inspection of the edge of the painting. The presence of a double-canvas support is often indicative of a remounting and/or restoration of the painting. We also note what appear to be mass-produced wire nails in the stretcher supporting a possible late 19th or 20th century remounting, but no record exists of this. A few pronounced inhomegeneities inside the canvas, which produce additional echoes, are evident in the B-scan and in the C-scan based on the peak-to-valley value in the time slice, in Fig. 2(c) above.

Moving above the canvas into the paint layers (optical delay <14 ps), however, the THz B-scans based on the raw data do not provide any clear stratigraphic detail, since the stratigraphy of the painting above the canvas is hidden in the first cycle of the reflected THz signal (which is blocked in yellow in Fig. 3), and the depth resolution based on the raw THz signals is not high enough to resolve the various paint layers. The available THz spectrum in our system extends to ~3 THz; this corresponds to ~100 *μ*m. Accounting for a representative refractive index of 1.85^10^, this limits us to a depth resolution, conventionally defined, of 100 *μ*m/1.85 ≈ 54 *μ*m. In other words, the absence of useful information from the raw THz is not merely due to the overlapping echoes; the minimum optical thickness that can be resolved is limited to ~100 *μ*m. Nonetheless, as we shall see, due to the nature of the painting’s layer structure, we can reconstruct the stratigraphy on a scale significantly below ~100 *μ*m. This is also the reason the time-domain THz C-scan in Fig. 2(a) contains both the surface and subsurface features.

### Sparsity-based Deconvolved Signals

**Figure 4:**
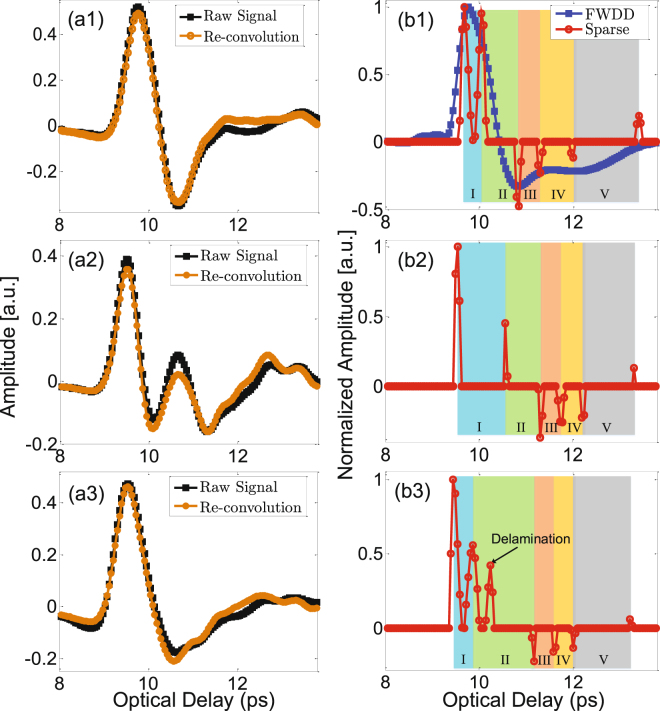
Typical THz reflected signals and the sparsity-based deconvolved signals. Figure 4(a1–a3) show examples of raw reflected signals (black) and signals reconstructed (brown) from the sparse-deconvolution and reference signals. Figure 4(b1–b3) show the corresponding sparsity-based deconvolution signals (red); Fig. 4(b1) also shows the deconvolved signal achieved by FWDD for comparison.

As mentioned above, the detailed stratigraphy associated with the painting itself is not evident in the raw data. Nonetheless, the sought for information is contained in the data; it is a matter how to extract it in order to reconstruct the stratigraphy. Sparsity-based time-domain deconvolution based on the shrinkage algorithm is utilized to process the 3D volume raw data. After deconvolution, a sparsity-based impulse-response function is achieved, which entirely depends on the stratigraphy and provides a new imaging domain with enhanced depth resolution. In the left-hand frames are shown three examples of raw THz signals received at various pixels (black curves) in Fig. 4(a1–a3). The corresponding sparsity-based impulse response functions are shown in the right-hand frames, Fig. 4(b1–b3). FWDD (detailed procedure in^26^) is also employed to process the raw data for comparison. In the deconvolution process, we consider the THz reference signal as the input and the reflected THz signal as the output; therefore, the actual impulse-response function associated with reflection coefficients should be obtained by multiplying the deconvolved signal by a factor of −1 for phase correction.

The sparsity-based deconvolution result of the reflected THz signal at pixel (89, 150) is shown in Fig. 4(b1). Six peaks are clearly identified in the sparsity-based impulse-response function, which correspond to five separated layers above the canvas. Compared with the result achieved by FWDD, which presents quite broad peaks and fails to reveal the underlying stratigraphy, the sparsity-based deconvolution result shows sharp features associated with interfaces between optically thin layers. Based on a knowledge of the typical structure of easel paintings^27^ of this period and visual inspection (see below), these five layers are expected to correspond to the varnish, pictorial layer, underpainting layer, imprimatura, and ground layer. The peaks are positive or negative, depending on whether the refractive index of the following layer is larger or less than that of the preceding layer. Finally, to check that the sparsity-based deconvolved signals indeed correspond to our raw signals, we convolve the deconvolved signals with the THz reference signal obtained by reflecting off a metal plate, which is referred to as “re-convolution”, and recover reconstructed signals [brown curves, Fig. 4(a1–a3)] with high fidelity compared with the received raw signals. This verifies our sparsity-based deconvolution procedure.

**Figure 5:**
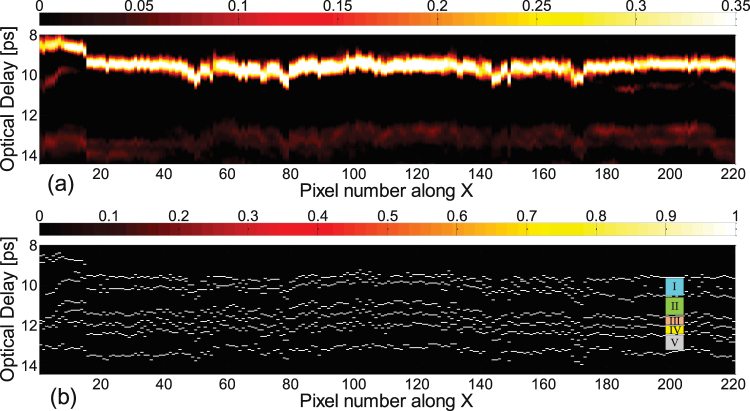
Comparison for optical delay corresponding to the layers above the canvas between (**a**) the THz B-scan based on the raw data (Fig. 3 on an expanded vertical scale) and (**b**) the binary THz B-scan based on the deconvolved data with the cross-section *Y* = 150 in which a valid peak is assigned value ‘1’ and the other positions ‘0’ regardless of the sign or height of the peak. I: varnish; II: pictorial layer; III: underpainting; IV: imprimatura; V: ground layer.

The sparsity-based impulse-response function, which consists of a baseline at zero and then a sequence of sharp peaks, enables us to reconstruct the detailed stratigraphy of the painting. By performing a peak-detection (both positive and negative peaks), binary THz B-scans, in which a valid peak is assigned value ‘1’ and the other positions ‘0’ regardless of the sign or height of the peak, can be obtained. The binary THz B-scan with the cross-section *Y* = 150 is shown in Fig. 5(b). The comparison with the THz B-scan based on the raw signals in Fig. 5(a), shows that the binary THz B-scan unlocks a wealth of information absent in the raw B-scan. It reveals, for the first time based on THz reflectometry, the detailed stratigraphy above the supporting canvas of a pre-19th century easel painting.

### Reconstruction of the Stratigraphy

In this section we synthesize our foregoing results to provide a tentative account of the stratigraphy of *Madonna in Preghiera*. The sparsity-based deconvolved signal, as seen above, contains a wealth of information concerning the various layers present in the painting as seen in the B-scan of Fig. 5. In order to reconstruct the layer-structure *across* the painting, it is useful to present the deconvolved data as C-scans that emphasize individual layers. This can be done by focusing on the amplitude of a specific reflected peak or the optical delay between specific successive peaks in the signals obtained across the canvas.

**Figure 6:**
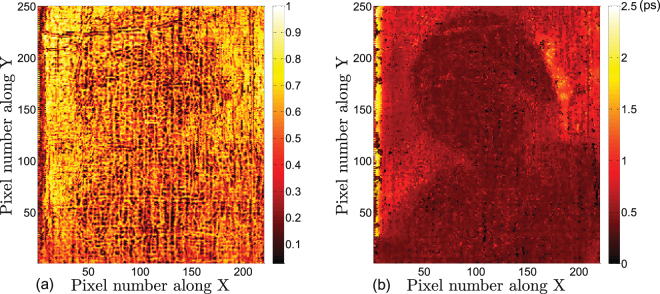
Varnish features revealed by THz sparsity-based deconvolved signals. (**a**) THz C-scan based on the amplitude of the first peak of the deconvolved signal. The contrast is normalized to one; (**b**) THz C-scan based on the optical delay between the first and second peaks of the deconvolved signal.

THz C-scans based on the amplitude of the first reflected peak and C-scans based on the optical delay between the first and the second peaks in the deconvolved signals are shown in Fig. 6, which reveal the features of the topmost layer in the painting, *viz*, the varnish. The THz C-scan in Fig. 6(a), shows only faint features associated with the painting’s composition. Instead, the C-scan is dominated by the morphology of the varnish surface, *i*.*e*., the surface roughness and variations associated with the craquelure due to aging. The lighter, more yellow areas to the left and right of the *Madonna*’s head indicate a smoother surface morphology in these regions. The THz C-scan in Fig. 6(b), based on optical delay between the first two reflected peaks, provides the quantitative information about the varnish *thickness* across the painting. Surface-morphology-related features are somewhat suppressed as they do not have a major effect on the varnish thickness across the painting. Based on this C-scan, we observe that the varnish around the *Madonna*’s profile is thicker to the left and right of her head--the same areas indicating a higher-amplitude first reflected peak. Another striking point is that a small region near the right of the *Madonna*’s head, as well as the left edge of the painting, shows much thicker varnish. Note that these features seen in the two C-scans discussed above are also evident in the raw and sparsity-based deconvolved signals. At representative pixel (185,158), the optical delay between the first and second peak is larger, as shown in Fig. 4(b2).

**Figure 7:**
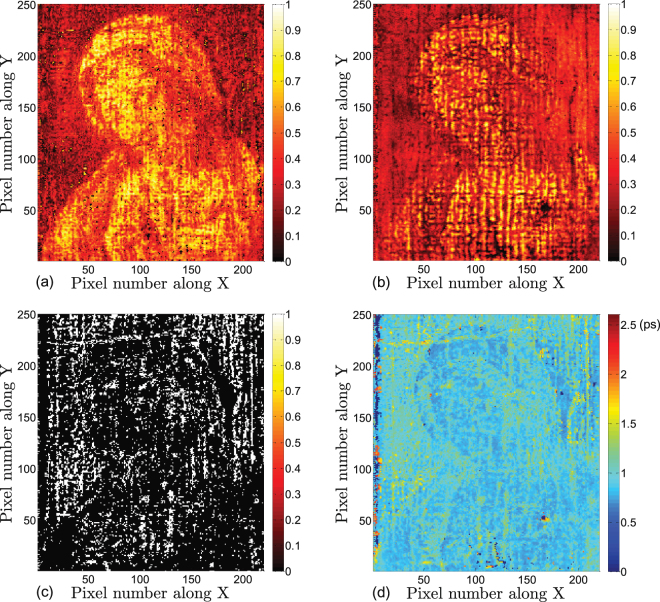
Features of the pictorial layer revealed by THz sparsity-based deconvolved signals. (**a**) THz C-scan based on the amplitude of second peak of the deconvolved signals; (**b**) THz C-scan based on the amplitude of the peak corresponding to the interface between the pictorial and underpainting layers; (**c**) binary THz C-scan indicating the positions where delamination occurs; (**d**) THz C-scan based on the optical delay between the peaks corresponding to the thickness of the pictorial layer.

The THz C-scans based on the amplitudes of the second and third peaks, corresponding to the varnish/pictorial and pictorial/underpainting interfaces, are plotted in Fig. 7(a,b). On the one hand, Fig. 7(a) clearly shows the painting’s composition, and is due to the pigment-dependent refractive index of the various colors evident in the final composition. On the other hand, in Fig. 7(b) the composition, while visible is less pronounced than in Fig. 7(a). This is due to the likelihood that the first-applied pictorial layer may have been fairly uniform in color as was common practice; the most prominent features of the composition, such as drapery, facial features, and hands, were painted subsequently. The various individual paint applications in the pictorial layer thus involve fairly gradual changes in refractive index in layers too thin to resolve in our measurements. Also note that a fine network of horizontal and vertical lines is evident. The optical delay between the second and third peaks in the deconvolved signals provides information about the optical thickness of the pictorial layer; however, the features are not uniform across the painting, shown in Fig. 7(d), and the horizontal and vertical lines are more pronounced. These lines are associated with the age-induced craquelure, where an addition peak between the second and the third peaks can be detected in the corresponding sparsity-based deconvolved signal. A typical signal illustrating this at pixel (112, 229) (a typical pixel in the horizontal line) is shown in Fig. 4(a3,b3). The physical origin of this peak is the existence of an additional air gap in these areas; however, this layer is too thin to be resolved even in the deconvolved signal, which generates only one observable positive peak. We summarize the presence of this additional peak, and thus delaminated regions, in the binary THz C-scan in Fig. 7(c). In this image, white regions indicate the presence of the additional peak, black its absence. The white regions are highly correlated with the craquelure; this is not entirely surprising, since stresses associated with craquelure formation and the channels open to the infiltration of moisture and other contaminants may also lead to delamination localized there.

**Figure 8:**
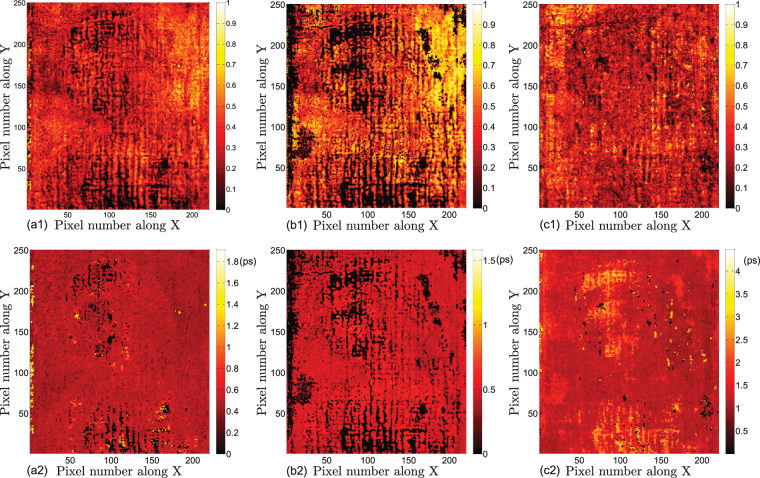
Features of the underpainting, imprimatura, and ground layers revealed by THz C-scans based on sparsity-based deconvolved signals. THz C-scans based on the amplitude of (**a1**) the fourth, (**b1**) fifth, and (**c1**) sixth peak. Contrast is normalized to one. THz C-scans based on the optical delay between (**a2**) the third and fourth peaks, (**b2**) fourth and fifth peaks, and (**c2**) fifth and sixth peaks.

Continuing to consider subsequent peaks in the sparsity-based deconvolved signal, features associated with the underpainting, imprimatura, and ground layers are investigated based on the THz C-scans in Fig. 8. Figure 8(a1–a3) provide C-scans based on the amplitudes of the fourth, fifth, and sixth peaks, respectively. Lines associated with the craquelure are still evident due to the shadow effects associated with reduced signals reaching subsequent layers depending on THz reflectivity and scattering from layers above. We note that the fourth and fifth peaks cannot be resolved in the deconvolved signals in all the pixels across the painting, corresponding to the black regions in the THz C-scans. Most of the black regions appear in the woman’s profile. This might be due to the multiple paint applications in the profile, which makes the layers underneath thinner than the depth resolution achieved. In Fig. 8(b1–b3) are shown C-scans computed from the optical delay between the third and fourth, the fourth and fifth, and the fifth and sixth peaks, respectively, if resolved. We note that, for Fig. 8(c), the optical delay is calculated based on the peak corresponding to the ground/canvas interface and the preceding peak which can be resolved. Based on the results, we conclude that the Master of the *Madonna in Preghiera* applied layers of fairly uniform thickness.

## Discussion

**Figure 9:**
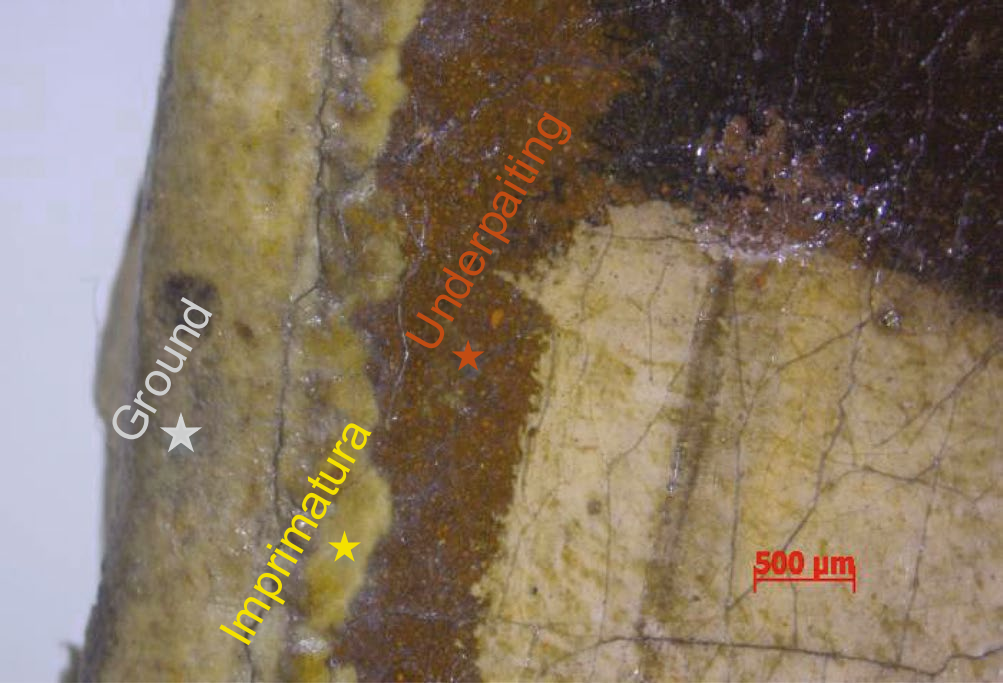
The optical microscopic image of a small region at the edge of *Madonna in Preghiera*. The applications of ground, imprimatura, and the underpainting can be clearly identified.

Sparsity-based THz reflectometry provides detailed 3D information on the 10-*μm*-to-*mm* scale in the depth direction and the 100-of-*μm*-to-*m* scale in the transverse directions, enabling us to reconstruct the detailed stratigraphy of the *Madonna in Preghiera*. Quantitative information in depth, such as the physical thicknesses of each resolved layer, can be estimated based on the knowledge of corresponding refractive indices. Starting with the canvas, a ground layer (gesso) is applied to seal the canvas and to create a smooth surface on which to paint. The imprimatura, literally the ‘first paint layer,’ follows the ground that would seal the oil-absorbent layer. Without the imprimatura, paint directly applied would soak into the ground and be difficult to control. The underpainting layer provides a proper foundation of the scene/subject matter, which was typically painted in a dark and muted monochrome tone, usually consisting of umber, as brown underpainting has often be used in oil painting from the 15th to 17th centuries. Such an approach was widespread to the point that the entire tonality and compositions of paintings frequently accounted for this dark underpainting. THz B- and C-scans clearly reveal the features of the ground, imprimatura, and the underpainting layers, which are fairly uniform across the painting. Assuming the refractive index of gesso is about 1.52^10^, the average physical thickness of the ground layer is about 132.7 *μ*m. For other oil-based paint layers, although the difference of refractive index between each layer is sufficient to produce the THz reflections, we assume a mean refractive index of 1.85^10^ in order to estimate the physical thickness of each paint layer. Based on this assumed refractive index, we estimate the average thickness of imprimatura is ~34 *μ*m and the average thickness of the underpainting layer is ~38 *μ*m. The presence of these three preparatory layers is confirmed by optical microscopy near the painting’s edge, shown in Fig. 9, where these layers are exposed.

**Figure 10:**
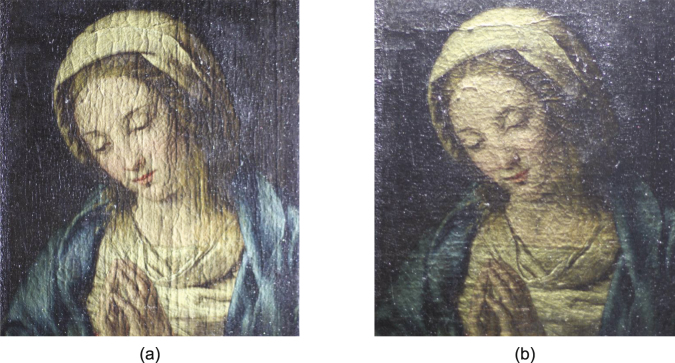
White-light raking images of *Madonna in Preghiera* with the light source (**a**) placed on the right side and (**b**) the top side of the painting.

The pictorial layers are applied subsequent to the underpainting. They contain the visually evident composition of the finished painting, and consist of additional applications of background color, the figure of the *Madonna*, facial features, hands, the gossamer veil, and other fine details. In the THz C-scans, textural features due to the craquelure first become pronounced in the pictorial layer. This is also where we observe the presence of delamination, closely associated with the craquelure. The surface morphology due to the craquelure can also be seen in white-light raking images shown in Fig. 10. In Fig. 10(a), the raking light source was placed to the right of the painting, emphasizing inhomogeneities oriented in the vertical direction; in Fig. 10(b), the raking light source was placed toward the top side of the painting, emphasizing inhomogeneities oriented in the horizontal direction. Again, there is strong correlation of the features seen in the raking-light images and in the THz C-scans in Fig. 7. Based on the assumed refractive index 1.85, the average physical thickness of the pictorial layer is about 64 *μm* in the regions without craquelure.

It was typical in the 17th century finally to apply varnish (a natural resin) to saturate the paint colors and protect the surface. The craquelure continues to be pronounced in the varnish, as is evidenced in the corresponding THz C-scans. The application of the varnish is not uniform across the painting. Assuming the refractive index of the varnish is about 1.6^28^, the physical thickness of the varnish at pixel (89,150) is about 35 *μ*m. It is important to note that the thicker region near the *Madonna*’s head is clearly identified, which exhibits the same shape as the dark areas in the UV fluorescence image in Fig. 1(c). The physical thickness at pixel (185,158) is estimated as 96 *μm*. This thick region is identified as a retouchings of the varnish. Visual inspection shows that these regions exhibit an anomalous texture.

The depth resolution of THz reflectometry is greatly enhanced by exploiting sparsity-based deconvolution. Indeed, conventional deconvolution methods, such as FWDD, entirely fail to reveal the underlying stratigraphy in easel paintings, because FWDD actually involves filtering out low- and high-frequency noise introduced from the division operation in the conventional deconvolution process, and therefore, narrows the bandwidth of the resulting impulse-response function^29^. Sparsity-based deconvolution is a pure time-domain method which does not introduce the low- and high-frequency noise, and is able to yield an enhanced depth resolution and achieve a more clear representation of the stratigraphy. In addition, sparsity-based deconvolution appears to have a better dynamic range than FWDD which implies that it is more effective in resolving small echoes. This property is especially important, since the difference of refractive index between various layers is often small in art paintings. We note that the depth resolution achieved by sparsity-based deconvolution is fundamentally limited by the coherence length of the THz source and the signal-to-noise ratio during the measurement.

In summary, sparsity-based THz reflectometry presented in this study clearly revealed the detailed stratigraphy of a 17th century easel painting with layer-thicknesses less than 50 *μ*m, including the varnish, pictorial, underpainting, imprimatura, and the ground layers across the entire painting. Retouching of varnish, as well as age-induced craquelure in the pictorial layer are also successfully characterized. The results achieved by sparsity-based THz reflectometry are supported by other techniques. We have demonstrated that sparsity-based THz reflectometry promises to provide an effective *in*-*situ* 3D quantitative imaging modality with high depth resolution and dynamic range for a broad range of cultural heritage objects, and an invaluable contribution to art-historical studies, as well as for conservation, restoration, and authentication.

## Methods

### THz reflective imaging

A typical THz time-domain system (Teraview TPS Spectra 3000) is employed in this study. The GaAs photoconductive antenna is excited by an ultrafast laser to produce roughly single-cycle THz pulses with bandwidth extending from 60 GHz to 3 THz. The maximum peak of its power spectrum is located at about 0.3 THz. The focus spot size of the THz beam is frequency-dependent, and is about 300 *μm* at 1 THz. Each recorded temporal reflected THz waveform contains 1024 data points, and the data sampling period is set to 0.0465 *ps*. The signal is averaged over 10 shots per pixel to enhance signal to noise. The scanning of the painting was conducted in a temperature-controlled laboratory at 22 °C. The humidity in the laboratory was held about 38%.

### Sparsity-based Time-Domain Deconvolution

In the time domain, the THz reflected signal (electric field) *r*(*t*) is the convolution of the incident THz pulse *i*(*t*) with the impulse-response function *h*(*t*), which corresponds to the structure and properties of the sample at a given point of interest,

1 $$r(t)=i(t)\otimes h(t)={\int }_{-\infty }^{+\infty }\,i(\tau )h(t-\tau )d\tau .$$

For reflective THz imaging, the incident THz pulse *i*(*t*) can be obtained by first recording the THz signal reflected from a metal plate (THz reference signal), and then multiplying the reference signal by a factor of −1 for phase correction. In practice, we should consider the discrete form of (1) with the sampling period *T*_*s*_,

2 $${r}_{n}=\sum _{m=0}^{M-1}\,{i}_{m}{h}_{n-m}+{e}_{n},$$

where *r*_*n*_ = *r*(*nT*_*s*_), *i*_*m*_ = *i*(*mT*_*s*_) and *e*_*n*_ accounts for the noise originating from the measurement system and materials with *n* and *m* as the indices of data points, and *M* as the length of the data points. Let column vectors **r**, **i**, **h** and **e** collect the samples of *r*_*n*_, *i*_*n*_, *h*_*n*_ and *e*_*n*_, respectively. Then (2) can be expressed as

3 $${\bf{r}}={\bf{Ah}}+{\bf{e}},$$

where **A** is the convolution matrix whose columns are delayed versions of **i**.

The basic idea of sparse deconvolution is to achieve the impulse response function by exploiting the sparse constraint. It aims at approximating the received THz signal **r** with **Ah** where **h** is a sparse sequence; that is, **h** has only few non-zero components. In this case, the sparse vector **h** can be computed by solving the *l*_0_ regularized optimization problem, which is defined as

4 $$\mathop{{\rm{\min }}}\limits_{{\bf{h}}}\,\frac{1}{2}{\Vert {\bf{Ah}}-{\bf{r}}\Vert }_{2}^{2}+\lambda {\Vert {\bf{h}}\Vert }_{0},$$

where ${\Vert {\bf{h}}\Vert }_{0}$ is the *l*_0_-norm of **h**, which is defined to be the number of nonzero entries in **h**, and *λ* is the regularization parameter, which controls the tradeoff between the sparsity of **h** and the residue norm. However, solving the non-convex *l*_0_ regularized optimization problem is known to be nonpolynomial hard and the global optimum cannot be guaranteed. It has already been shown that this non-convex optimization problem can be approximated with a convex optimization problem by replacing the *l*_0_ penalty with the *l*_1_ penalty as

5 $$\mathop{{\rm{\min }}}\limits_{{\bf{h}}}\,\frac{1}{2}{\Vert {\bf{Ah}}-{\bf{r}}\Vert }_{2}^{2}+\lambda {\Vert {\bf{h}}\Vert }_{1},$$

where ${\Vert {\bf{h}}\Vert }_{1}$ is the *l*_1_-norm of **h**, which is defined as the sum of the absolute values of its components. Since the *l*_1_-norm is convex, a global optimum can be guaranteed. An iterative shrinkage algorithm is utilized in this study because of its effectiveness and the limited number of parameters that need to be tuned. The general iterative procedure is given by:

6 $${{\bf{h}}}_{i+1}={S}_{\lambda \tau }({{\bf{h}}}_{i}-\tau {{\bf{A}}}^{{\rm{T}}}({\bf{A}}{{\bf{h}}}_{i}-{\bf{r}})),$$

where *τ* is an appropriate step size, which should obey

7 $$\tau  < \frac{2}{{\Vert {{\bf{A}}}^{{\rm{T}}}{\bf{A}}\Vert }_{2}},$$

in order to guarantee convergence, and the shrinkage or soft-thresholding operator *S*_*λτ*_ is defined as

8 $${S}_{\lambda \tau }(h[n])=\{\begin{array}{ll}h[n]+\lambda \tau  & h[n]\le -\lambda \tau \\ 0 & |h[n]| < \lambda \tau \\ h[n]-\lambda \tau  & h[n]\ge \lambda \tau \end{array}.$$

A thorough theoretical analysis^30^ proves the convergence of this iterative shrinkage algorithm guaranteeing that the solution is the global minimizer for convex **h**. For processing the raw 3D volume THz data from the painting in this study, the iteration based on Eq. 6 was performed 3000 times with the regulation parameter *λ* = 0.4 and the step size ${1.2/\Vert {{\bf{A}}}^{{\rm{T}}}{\bf{A}}\Vert }_{2}$. Detailed information on the accuracy of the algorithm can be found in our previous results^24^.

### Data Availability

The datasets generated during and/or analysed during the current study are available from the corresponding author on reasonable request.
